# Chemical Profiling of Latvian Propolis: Regional Variations and Botanical Origins

**DOI:** 10.3390/molecules30234533

**Published:** 2025-11-24

**Authors:** Freideriki Papakosta, Konstantia Graikou, Evgenia Panou, Fani Hatjina, Leonidas Charistos, Valters Brusbardis, Josef J. M. van der Steen, Ioanna Chinou

**Affiliations:** 1 Laboratory of Pharmacognosy and Chemistry of Natural Products, Faculty of Pharmacy, National and Kapodistrian University of Athens, 15771 Athens, Greece; freiderikipa@pharm.uoa.gr (F.P.); kgraikou@pharm.uoa.gr (K.G.); evpanou@pharm.uoa.gr (E.P.); 2 Department of Apiculture, Institute of Animal Science, Hellenic Agricultural Organization—DIMITRA (ELGO-DIMITRA), 11145 Nea Moudania, Greece; fhatjina@gmail.com (F.H.); leocharistos@elgo.gr (L.C.); 3 Latvian Beekeepers Association, Rigas iela 22, LV-3004 Jelgava, Latvia; valters@strops.lv; 4 Alveus AB Consultancy, Kerkstraat 96, 5061 EL Oisterwijk, The Netherlands; alveusab@outlook.com

**Keywords:** propolis, Latvia, GC-MS, phenolic acids, flavonoids, European-type propolis

## Abstract

Propolis is a resinous natural product produced by honeybees from plant exudates and beeswax. Its complex chemical composition varies significantly with geographical origin and seasonal factors. This study aimed to characterize the chemical composition of propolis samples collected from different regions of Latvia using gas chromatography–mass spectrometry (GC-MS). In total, 47 metabolites were identified, with chemical profiles dominated by phenolic acids and their esters—compounds typical of European propolis. Regional variations reflected differences in local vegetation, and two samples exhibited flavonoid constituents indicative of poplar-type botanical sources. The Daugavpils sample, which showed a particularly rich chemical profile, was further fractionated chromatographically, leading to the isolation of twelve metabolites: 2’,4’,6’-trihydroxy-4-methoxy dihydrochalcone, 2’,6’,4-trihydroxy-4’-methoxy dihydrochalcone, 2’,6’-dihydroxy-4,4’-dimethoxy dihydrochalcone, 2’,6’-dihydroxy-4’-methoxy dihydrochalcone, 2’,4’,6’-trihydroxy dihydrochalcone, palmitic acid, benzyl benzoate, cinnamyl cinnamate, pinostrobin, pinostrobin chalcone, pinocembrin, and pinobanksin. Given the limited prior research on Latvian propolis, this study provides valuable insights into its chemical diversity and the influence of regional flora on its composition.

## 1. Introduction

Propolis is a resinous product produced by honeybees, collected from plant buds, exudates, or resins found on the stems, branches, and leaves of various plant species. The chemical composition of raw propolis typically consists of approximately 50% plant resins, 30% waxes, 10% essential and aromatic oils, 5% pollen, and 5% other organic compounds [1]. Bees use this natural material as a defensive substance within the hive—sealing cracks, maintaining stable temperature and humidity, and creating an antiseptic environment that protects against microbial infections. Because propolis is gathered from a wide variety of plants, its chemical composition varies significantly depending on the geographic region, climate, local flora, and season of collection [2,3]. More than 850 chemical constituents have been identified in propolis samples worldwide, including flavonoids, terpenes, and phenolic compounds [4].

Several types of propolis have been described based on their geographic origin, such as temperate, tropical, birch, Mediterranean, and Pacific propolis [5]. However, classifying propolis based on its botanical source has been suggested as a crucial initial step in quality control, as this approach defines the specific compounds that should be quantitatively monitored as the main bioactive constituents [6]. The main plant sources of propolis in the boreal and temperate regions of Europe are the bud resins of black poplar (*Populus nigra*), downy birch (*Betula pubescens*), and common aspen (*Populus tremula*) [7]. Chemical markers have been identified for each of these resins. Black poplar bud resin is rich in phenolic compounds, including pentenyl (mainly prenyl) cinnamates, chalcones, and unsubstituted B-ring flavonoids such as pinocembrin, pinostrobin, chrysin, galangin, pinobanksin, and their 3-substituted derivatives. In contrast, downy birch and common aspen bud resins are characterized by distinct phenylpropenoids, i.e., hydroxycinnamic acid esters of sesquiterpene alcohols and glycerol, respectively [7]. Although bees from a single colony may collect resins from multiple tree species, European propolis can be classified into these three main types—poplar, birch, and aspen type—based on their species-specific chemical profiles [8,9,10]. Mixed types of propolis containing exudates of more than one plant species have also been reported [6,9].

Latvia lies at the intersection of the boreal and hemi-boreal vegetation zones, creating a unique botanical landscape with a mixture of northern birch–aspen forests and temperate deciduous species. This transitional flora, combined with Latvia’s cooler, humid maritime–continental climate, may influence the resin sources available to bees and result in a chemical profile that differs from that of Europe.

In recent years, research has increasingly focused not only on the chemical composition of propolis but also on the identification and biological efficacy of its bioactive compounds. Traditionally used in folk medicine, propolis is well known for its antimicrobial, anti-inflammatory, and antioxidant activities, which contribute to a broad range of potential health benefits [11].

Latvia is located in the Baltic region of Northern Europe with its capital city in Riga. The country encompasses a diverse landscape, including agricultural land, extensive forest areas, and numerous inland water bodies. Most of the country consists of fertile lowland plains with an abundance of forests where pine, birch, and spruce are mainly found. Several flora species from Latvia are recognized as a national symbol such as the oak (*Quercus robur*), the linden (*Tilia cordata*), and the white anthurium (*Leucanthemum vulgare*) [12]. The country is characterized by a temperate and cold climate, with average winter temperatures around −6 °C and summer temperatures averaging 19 °C. This climatic pattern, combined with Latvia’s northern geographic position, is clearly reflected in its native flora.

Latvia is classified into eight distinct geobotanical regions, each of which presents unique vegetation types shaped by various factors: climatic, geological, soil, and landscape [13]. The Coastal geobotanical region shows the highest percentage of forest cover (60–70% of the geobotanical area). In contrast, Kurzeme in Western Latvia has less than 40% forest cover and is the only region with *Carpinus betulus* forests. The Zemgale geobotanical region is extensively cultivated with agricultural lands covering 76% of the area, with deciduous tree forests, mainly *Fraxinus excelsior*. The Central Latvia geobotanical region is rich in forests, which cover approximately 55–62% with widespread *Pinus sylvestris* and *Picea abies* species. The North Vidzeme geobotanical region and the Northeastern geobotanical region include plains, while North Vidzeme also has a wide variety of broadleaf deciduous trees. The Central and Southeastern Vidzeme are located at higher altitudes with the presence of spruce forests and agricultural lands [14].

The natural conditions in Latvia are highly conducive to beekeeping, which is considered a classic branch of agriculture; however, there is no specific traditional beekeeping region, as this practice is widespread across all areas of the country. The only species of bee utilized in Latvia is *Apis mellifera* [7,10], and due to the rich diversity of flora present in the country, the nectar collected by bees is correspondingly varied [15]. Furthermore, the practice of precision beekeeping recently emerged and is continuing to develop in Latvia [16], enhancing the management and productivity of beekeeping operations.

The growing interest in propolis has prompted researchers worldwide to investigate its chemical composition. To date, chemical analyses of Latvian propolis have been limited: only propolis samples from the northeastern region, specifically the Tirza area, have undergone chemical analysis using GC-MS [7,10], while studies have also reported on the phenolic acid content of propolis from the Riga region through HPLC analysis [17]. Consequently, the present study aimed to conduct a comprehensive chemical analysis of propolis samples from various geographical regions of Latvia providing an overall assessment of Latvian propolis and enabling the isolation and identification of its most abundant metabolites.

## 2. Results

### 2.1. Propolis Composition

#### GC-MS Analysis

**Figure 1:**
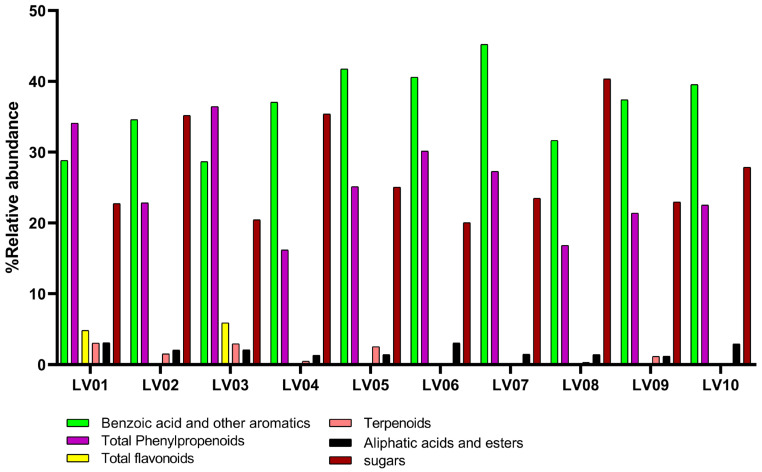
Main chemical categories of compounds from the studied propolis samples of Latvia. Categories include benzoic acid and other aromatics, total phenylpropanoids, total flavonoids, terpenoids, aliphatic acids and esters, sugars. Relative abundances are shown for each sample, highlighting regional differences between LV01: Jelgava, LV02: Riga, LV03: Daugavpils, LV04: Rujiena, LV05: Balvi, LV06: Aizpute, LV07: Svete, LV08: Ragana, LV09: Talsi, LV10: Cesis.

The chemical composition of ten Latvian propolis samples (LV01–LV10) from different geographic regions (70% ethanolic extracts) was analyzed by GC-MS following silylation, leading to the identification of 47 individual compounds (Table S1). Their main chemical categories are summarized in Table 1 and Figure 1. According to the chemical classification, the analysis demonstrated the presence of phenolic acids (mainly benzoic, p-coumaric, isoferulic, and caffeic acid and their derivatives) in all studied samples, while flavonoids and chalcones (such as pinocembrin, pinobanksin, as well as pinostrobin chalcone and dihydrochalcones) were identified only in LV01, LV02, and LV03.

### 2.2. Isolation of Chemical Constituents

**Figure 2:**
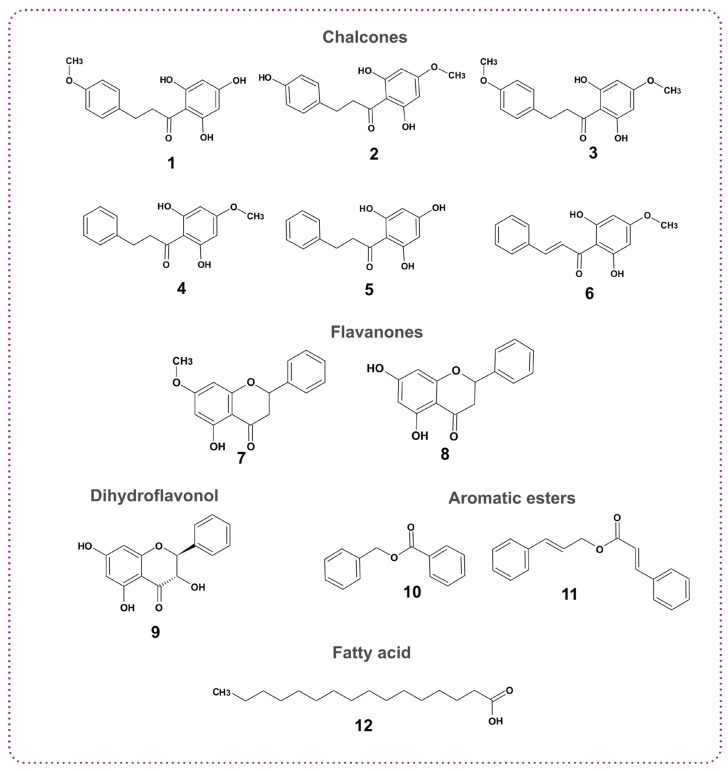
The isolated compounds from propolis sample LV03; **1**: 2’,4’,6’-trihydroxy-4-methoxy-dihydrochalcone; **2**: 2’,6’,4-trihydroxy-4’-methoxy-dihydrochalcone; **3**: 2’,6’-dihydroxy-4,4’-dimethoxy-dihydrochalcone; **4**: 2’,6’-dihydroxy-4’-methoxy-dihydrochalcone; **5**: 2’,4’,6’-trihydroxydihydrochalcone, **6**: pinostrobin chalcone; **7:** pinostrobin; **8**: pinocembrin; **9**: pinobanksin; **10**: benzyl benzoate; **11**: cinnamyl cinnamate; **12**: palmitic acid.

In this study, further analysis was performed on the sample LV03, due to its high content of flavonoids (5.87%) and phenolic acids (65.09%). Twelve metabolites were isolated using various chromatographic techniques and identified through NMR spectral analysis, with their structures confirmed by comparison with the literature data [18,19,20,21,22,23,24,25,26,27]. These included nine flavonoids of which six were chalcones (2’,4’,6’-trihydroxy-4-methoxy-dihydrochalcone, 2’,6’,4-trihydroxy-4’-methoxy-dihydrochalcone, 2’,6’-dihydroxy-4,4’-dimethoxy-dihydrochalcone, 2’,6’-dihydroxy-4’-methoxy-dihydrochalcone, 2’,4’,6’-trihydroxydihydrochalcone, pinostrobin chalcone); two were flavanones (pinostrobin and pinocembrin); and one was a dihydroflavonol (pinobanksin). Additionally, one fatty acid (palmitic acid) and two aromatic esters (benzyl benzoate and cinnamyl cinnamate) were isolated and identified (Figure 2).

## 3. Discussion

The chemical profiles of the propolis samples, as determined by GC-MS analysis, reflect the flora of the surrounding area. Aromatic compounds, including benzoic and cinnamic acids and their esters, were detected in all samples. Flavonoids such as chalcones, flavanones, flavonols, and flavanolols were identified in three out of ten propolis samples. Among the aromatic compounds, benzoic acid was consistently present in every sample, comprising a significant proportion ranging from 22.24% to 33.04%. These results are generally consistent with previous analyses of Latvian propolis, where benzoic acid and other aromatic acids were detected at abundances of 7.4%, 15.7%, and 17.9% [10].

Cinnamic acid derivatives, including *cis*- and *trans*-*p*-coumaric, *cis*- and *trans*-ferulic, isoferulic, and caffeic acids, were detected in all ten Latvian propolis samples, with *trans*-*p*-coumaric acid being the predominant contributor, ranging from 8.07% (LVO4) to 14% (LV03). The cinnamic esters benzyl *p*-coumarate, coniferyl benzoate, and, at lower levels, benzyl cinnamate were also present in all samples. In contrast, pentenyl *p*-coumarate, 3-methyl-3-butenyl, and 3-methyl-2-butenyl (prenyl) caffeates were detected exclusively in samples LV01 and LV03. Black poplar bud resin is known to be rich in phenolic compounds, including pentenyl cinnamates, primarily prenyl derivatives [28,29], suggesting a closer botanical relationship of these two samples to poplar type.

Flavonoids were primarily detected in two samples (LV01 and LV03), while LV02 contained small amounts of the flavanone pinocembrin, which was also present in LV01 and LV03. The latter two samples additionally contained three chalcones (2’,6’-dihydroxy-4’-methoxy dihydrochalcone, 2’,4’,6’-trihydroxychalcone, and pinostrobin chalcone), the flavanonol pinobanksin, and its acetate, as well as the flavonol galangin. Overall, the total flavonoid content in LV01 and LV03 was 4.81% and 5.87%, respectively. As previously reported, chalcones and flavonoids lacking B-ring substitution, such as pinocembrin, galangin, and pinobanksin, characterize black poplar bud resins [29]. Although, pure poplar-type propolis contains significantly higher amounts of total flavonoids [6,7], their presence, along with high levels of hydroxycinnamic acids (accounting for 34.1% in LV01 and 36.44% in LV03), strongly suggests the presence of *Populus nigra* in the surrounding flora.

The two existing studies on Latvian propolis samples from northeast Latvia have reported phenylpropanoid glycerides characteristic of the aspen type, with concentrations ranging from 11.4% to 33.5%. Esters of coniferyl alcohol with hydroxycinnamic acids, also typical markers of this type, were detected in the same studied Latvian propolis at approximately 8% [7,10]. However, none of the samples analyzed in this study contained such conjugates, and consequently, none supported the proposed aspen-type propolis. The broad geographic sampling across Latvia in the current study, combined with previous research, captures the extensive chemical diversity and regional heterogeneity of Latvian propolis. These variations reflect notable chemical differences between geographical regions, likely shaped by bee foraging preferences influenced by the composition of the local flora.

Sesquiterpenoids were further found in LV01-LV05, LV08, and LV09, including caryophyllene oxide, *δ*-selinene, dehydroaromadendrene, and *α*- and *β*-eudesmol. Sesquiterpenes are dominant volatile compounds of volatile poplar propolis oils and are closely followed by non-terpenic aromatic compounds such as benzyl acetate, benzyl benzoate, and benzyl alcohol [29]. Although present in relatively low concentrations, their distinctive aroma and notable biological activity make these compounds important for propolis characterization, and volatile profiles can serve to differentiate propolis samples from various geographical regions [4].

3-Hydroxymyristic and 3-hydroxypalmitic acids, classified among the 3-hydroxy C14–C22 acids, were detected in sample LV01 (0.61%). Notably, this class of compounds, together with low flavonoid levels, is considered characteristic of the buds of *Aesculus hippocastanum* [10]. However, in bud resins of this species, they typically occur at higher levels of approximately 20% and are accompanied by significant amounts of triterpenoids, which were not detected in the LV samples, indicating only a minor contribution from this source.

According to Isidorov et al. [9], the primary plant sources of propolis in the temperate regions of Europe—characterized by a moderate climate, typically located between the Mediterranean (warm) and boreal (cold) zones—include areas of Eastern Europe such as parts of Ukraine, Belarus, and the Baltic States (Latvia, Lithuania, Estonia), with the main bud resins being from *Populus nigra* (black poplar type), *Betula pubescens* (downy birch type), and *Populus tremula* (aspen type). Additionally, research by Ristivojević et al. [29] has indicated that other genera such as Quercus, Ulmus, Picea, and *Fraxinus*, along with species like *Aesculus hippocastanum*, *Betula pendula*, *Salix alba*, *Alnus glutinosa*, and various *Pinus* species, serve as secondary sources of resinous secretions. The flora of Latvia supports the classification of propolis as European type, as it is dominated by deciduous trees such as *Carpinus betulus*, *Fraxinus excelsior*, *Pinus sylvestris*, *Picea abies*, *Acer pseudoplatanus*, *Betula pendula*, *Populus tremula*, *Quercus robur*, and *Acer platanoides* [14,30]. Additionally, variations within the European chemical type are observed depending on the local flora of each specific region.

It has already been stated that, when multiple resin sources are available to honeybee colonies, the composition of propolis rarely corresponds exclusively to a single plant precursor [10]. This is likely due to bees preferring resin diversity, which may offer enhanced protection against a range of pathogens through the synergistic effects of compounds derived from various plant materials [31]. Our findings align with this observation, as the chemical profiles of the analyzed propolis samples do not indicate the presence of a single dominant resin source. Instead, they suggest contributions from multiple botanical origins, reflecting a diverse resin foraging strategy by bees driven by the composition of the local flora.

Furthermore, sample LV03 from Daugavpils was subjected to chromatographic separations due to its rich profile of aromatic acids (65.09%) and the presence of flavonoids (5.87%). Twelve metabolites were isolated and structurally determined namely 2’,4’,6’-trihydroxy-4-methoxydihydrochalcone, 2’,6’,4-trihydroxy-4’ methoxydihydrochalcone, 2’,6’-dihydroxy-4,4’-dimethoxydihydrochalcone, 2’,6’-dihydroxy-4’-methoxy dihydrochalcone, 2’,4’,6’-trihydroxydihydrochalcone, palmitic acid, benzyl benzoate, cinnamyl cinnamate, pinostrobin, pinostrobin chalcone, pinocembrin and pinobanksin.

The five identified dihydrochalcones have been previously isolated from buds of *Populus balsamifera* [20] and have been also identified in propolis samples from Canada [32]; Northern California and Oregon, USA [33]; and England [34]. Benzyl benzoate and cinnamyl cinnamate are characteristic aromatic compounds in European-type propolis [29], with benzyl benzoate identified in Latvian propolis [10] and cinnamyl cinnamate isolated from Jordanian [35], Hondurian [36], Mexican [37], Brazilian [38], and Chinese propolis [39]. Pinocembrin, pinobanksin, pinostrobin, and pinostrobin chalcone, flavonoids without a substituted B ring, are typical constituents of poplar-type propolis [1,40], which have been isolated and identified in propolis samples from several countries [32,40,41,42,43]. Palmitic acid has been detected in many propolis samples, including those from Turkey [44], Jordan [43], Algeria [45], India [46], Canada [32], and Egypt [47].

## 4. Materials and Methods

### 4.1. Chemicals and Reagents

For column chromatography (CC), silica gel (Kieselgel 60 H Merck, Darmstadt, Germany) was used as the stationary phase, with gradient elution using the solvent mixtures indicated in each case. The solvents cyclohexane, dichloromethane (DCM), and methanol (MeOH) were of HPLC grade and were purchased from Fisher Chemical (Fisher Scientific, Loughborough, Leics, UK). Fractionation during all column chromatographic procedures was monitored by thin-layer chromatography (TLC) using Merck silica gel 60 F254 (0.2 mm layer thickness), Merck RP-18 F254S, and Merck cellulose plates (Merck, Darmstadt, Germany). For preparative thin-layer chromatography (prep TLC), silica gel 60 F254 (Merck, Darmstadt, Germany) was used. Detection on TLC plates was enabled using UV light (254 and 366 nm) and H_2_SO_4_–vanillin spray reagent on silica gel followed by heating.

For the extraction of the propolis samples, ethanol (Sigma-Aldrich, St. Louis, MO, USA) was used. For derivatization of the ethanolic extracts, dry pyridine (Prolabo, Paris, France) and N,O-bis(trimethylsilyl)trifluoroacetamide (BSTFA) (Merck, Darmstadt, Germany) were used.

### 4.2. Propolis Samples

**Figure 3:**
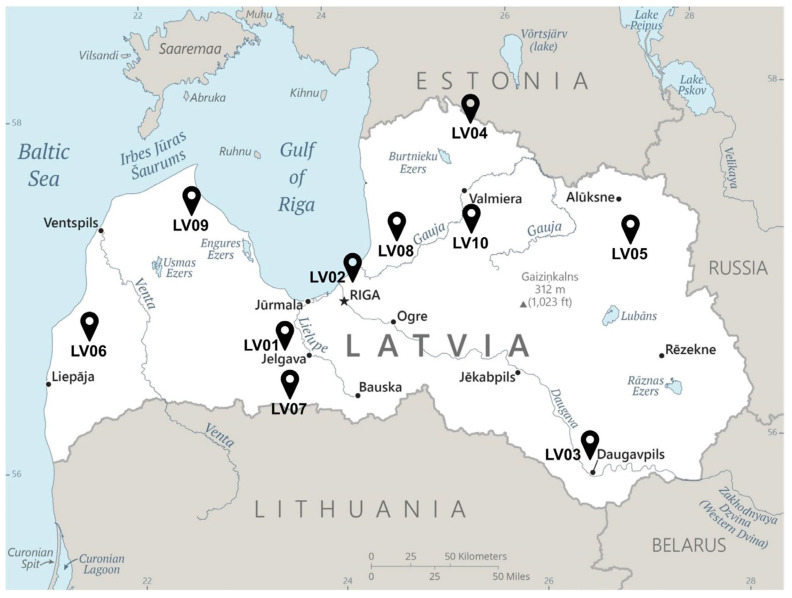
Map of Latvian propolis sample collection areas. Sample codes correspond to regions. (LV01: Jelgava, LV02: Riga, LV03: Daugavpils, LV04: Rujiena, LV05: Balvi, LV06: Aizpute, LV07: Svete, LV08: Ragana, LV09: Talsi, LV10: Cesis) [48].

Ten propolis samples were collected in July of 2022 from different regions of Latvia namely: Jelgava (LV01), Riga (LV02), Daugavpils (LV03), Rujiena (LV04), Balvi (LV05), Aizpute (LV06), Svete (LV07), Ragana (LV08), Talsi (LV09), and Cesis (LV10) (Figure 3, Table 2).

### 4.3. Extraction and Sample Preparation

Propolis samples (3 g) were extracted three times over a 24 h period using 70% ethanol at room temperature. Each extract was subsequently evaporated to dryness using a rotary evaporator at 40 °C. Approximately 5 mg of each dried extract was silylated with 40 μL of dry pyridine and 60 μL of bis(trimethylsilyl) trifluoroacetamide (BSTFA), heated at 80 °C for 20 min, and subsequently analyzed by GC-MS [49].

### 4.4. GC-MS Analysis

The component analysis was performed by the technique of gas chromatography coupled with mass spectrometry (gas chromatography–mass spectrometry, GC-MS). The analysis was conducted using an Agilent Technologies Gas Chromatograph 7820A connected to an Agilent Technologies 5977B mass spectrometer system based on electron impact (EI) and 70 eV of ionization energy. The gas chromatograph features a split/splitless injector and a 30 m long HP5MS capillary column with an internal diameter of 0.25 mm and a film thickness of 0.25 μm. The temperature program ramped from 100 °C to 300 °C at a rate of 5 °C per minute. Helium was used as the carrier gas at a flow rate of 0.7 mL/min, with an injection volume of 2 μL, a split ratio of 1:10, and an injector temperature set at 280 °C. The compounds were identified through computer searches utilizing the Wiley mass spectral databases, bibliographic information, and internal data. The components of the propolis extract were determined by considering their areas as a percentage of the total ion current. Each sample was analyzed in duplicate.

### 4.5. Fractionation and Purification Procedures of Ethanolic Extract

The ethanolic extract of LV03 (20 mg) was subjected to preparative TLC (silica plate DCM/MeOH 98/2), and five compounds were isolated and identified as 2’,4’,6’-trihydroxy-4-methoxy dihydrochalcone, 2’,6’,4-trihydroxy-4’-methoxy dihydrochalcone (asebogenin), 2’,6’-dihydroxy-4,4’-dimethoxy dihydrochalcone (calomelanone), 2’,6’-dihydroxy-4’-methoxy-dihydrochalcone, and 2’,4’,6’-trihydroxydihydrochalcone.

The ethanolic extract of LV03 (0.80 g) was further subjected to column chromatography with silica-60 gel column chromatography eluted with cyclohexane: DCM: MeOH (100:0:0–0:70:30) (gradient method) to afford 72 fractions (F1–F72). Fraction F2 (1.50 mg) was eluted with cyclohexane: DCM 80:20 and identified as palmitic acid. Fraction F5 (0.90 mg) was eluted with cyclohexane: DCM 70:30 and identified as benzyl benzoate. Fraction F8 (2.20 mg) was eluted with cyclohexane/DCM 70:30 and identified as cinnamyl cinnamate. Fraction F11 (4.20 mg) was eluted with cyclohexane: DCM 70:30 and identified as pinostrobin. Fraction F23 (0.80 mg) was eluted with cyclohexane: DCM 50:50 and identified as pinostrobin chalcone. Fraction F37 (0.70 mg) was eluted with cyclohexane: DCM 30:70 and identified as pinocembrin. Fraction F42 (10.40 mg) was eluted with cyclohexane: DCM 20:80 and identified as pinobanksin.

### 4.6. Nuclear Magnetic Resonance (NMR)

HSQC, HMBC were recorded on a Bruker Avance III 400 MHz (Bruker BioSpin, Rheinstetten, Germany) spectrometer using deuterated chloroform (CDCl3) (Eurisotop, Gif Sur Yvette, France) and methanol-d4 (CD3OD) (Eurisotop, Saint Aubin Cedex France). Chemical shifts are reported in ppm relative to the solvent signals [1H: δ (CDCl_3_) = 7.26 ppm; 13C: δ (CDCl_3_) = 77.20 ppm; 1H: δ (CD_3_OD) = 3.31 ppm; 13C: δ (CD_3_OD) = 49.10 ppm].

## 5. Conclusions

As propolis is recognized as an important health-promoting agent, the primary aim of this study was to conduct a chemical investigation of propolis samples from geographically diverse regions of Latvia for the first time. All analyzed propolis samples exhibited characteristics consistent with the European-type profile. They displayed a distinctive phenolic composition that is prevalent across Europe. This composition primarily includes phenolic acids, which are mainly derived from poplar trees (*Populus* spp.). Propolis samples from Daugavpils and Jelgava contained taxonomic markers of *Populus nigra* including flavonoids (5%) such as pinostrobin chalcone, pinocembrin, pinobanksin, pinobanksin 3-O-acetate, and galangin. These samples also contained a high concentration of phenolic acids. Propolis samples from the other regions, by contrast, were marked by the presence of phenolics and the absence of flavonoids. The chemical profile of the ethanolic extracts showed similarities with previously reported samples from northeastern Latvia, where phenolic derivatives predominate. However, phenylpropanoid glycerides, which are typical of the aspen type, were not detected in this study, highlighting the influence of botanical origin on propolis composition.

The wide geographic coverage in this study reveals the substantial chemical diversity and regional variation of Latvian propolis. These variations within the European chemical type are likely influenced by the unique local flora of each region. They are also coupled with bees’ preference for collecting resin from multiple botanical sources rather than from a single dominant one, resulting in distinct chemical profiles. Interestingly the findings suggest that, even in Europe, where propolis is generally considered well studied, surprising differences in plant origins and chemical composition can still be observed.

## Figures and Tables

**Table 1:** Chemical categories of propolis compounds from various regions of Latvia expressed as % relative abundance.

Chemical Category	LV01	LV02	LV03	LV04	LV05	LV06	LV07	LV08	LV09	LV10
Total phenylpropenoids including	34.1	22.84	36.44	16.2	25.13	30.16	27.29	16.82	21.38	22.53
Cinnamic acids	28.25	20.52	32.55	14.62	21.56	22.69	21.1	15.64	19.15	20.18
Cinnamic acid esters	5.85	2.32	3.89	1.58	3.57	7.47	6.19	1.18	2.23	2.35
Benzoic acid and other aromatics	28.84	34.59	28.65	37.07	41.76	40.59	45.23	31.66	37.4	39.56
Total flavonoids including	4.81	0.17	5.87							
Chalcones	1.46		3.25							
Flavanols	1.5		1.07							
Flavanones	1.02	0.17	1.11							
Flavonols	0.83		0.44							
Terpenoids including	3.04	1.52	2.94	0.48	2.53	0.28		0.31	1.15	
Sesquiterpenes	3.04	1.52	2.94	0.48	2.53			0.31	1.15	
Aliphatic acids and esters including	3.09	2.04	2.06	1.31	1.41	3.05	1.46	1.41	1.18	2.9
Aliphatic C12-C30 acids	2.48	2.04	2.06	1.31	1.41	3.05	1.46	1.41	1.18	2.9
3-Hydroxy C14-C22 acids	0.61									
Sugars	22.73	35.18	20.45	35.4	25.06	20.04	23.48	40.35	22.97	27.86

**Table 2:** Propolis collection areas from Latvia.

Sample	Collection Area
**LV0** **1**	Jelgava, Central Latvia
**LV0** **2**	Riga, North-Coastal Latvia
**LV0** **3**	Daugavpils, Southeast Latvia
**LV0** **4**	Rujiena, Northern Latvia
**LV0** **5**	Balvi, Eastern Latvia
**LV** **06**	Aizpute, Western Latvia
**LV** **07**	Svete, Southern Latvia
**LV** **08**	Ragana, North-Central Latvia
**LV** **09**	Talsi, Northwest Latvia
**LV** **10**	Cesis, Northeastern-Central Latvia

## Data Availability

The original contributions presented in this study are included in the article and Supplementary Materials, further inquiries can be directed to the corresponding author.

## Supplementary Materials

The following supporting information can be downloaded at https://www.mdpi.com/article/10.3390/molecules30234533/s1: Table S1: Chemical composition (% relative abundance) by GC-MS analysis of propolis samples from different geographic regions of Latvia.
