# Supplementary material for: Chemical Profiling of Latvian Propolis: Regional Variations and Botanical Origins
Source: Molecules. 2025 Nov 24;30(23):4533. doi: 10.3390/molecules30234533 (PMC12693659; doi:10.3390/molecules30234533)
Supplement: Supplementary file 1 [file molecules-30-04533-s001.zip › molecules-3997824-supplementary.pdf]

molecules

MDPI

Article

Chemical Profiling of Latvian Propolis: Regional Variations and Botanical Origins

Freideriki Papakosta[1], Konstantia Graikou[1], Evgenia Panou[1], Fani Hatjina[2], Leonidas Charistos[2], Valters Brusbardis[3], Josef J.M. van der Steen[4], Ioanna Chinou[1]*

[1] Laboratory of Pharmacognosy and Chemistry of Natural Products, Faculty of Pharmacy, National and Kapodistrian University of Athens, Panepistimiopolis, Zografou, 15771, Athens, Greece; freiderikipa@pharm.uoa.gr (F.P.); kgraikou@pharm.uoa.gr (K.G.); evpanou@pharm.uoa.gr (E.P.); ichinou@pharm.uoa.gr (I.C.).
[2] Department of Apiculture, Institute of Animal Science ELGO 'DIMITRA', 11145 Nea Moudania, Greece; fhatjina@gmail.com (F.H.); leocharistos@elgo.gr (L.C.).
[3] Latvian Beekeepers Association, Rigas iela 22, LV-3004 Jelgava Latvia; valters@strops.lv (V.B.)
[4] Alveus AB Consultancy, Kerkstraat 96, 5061 EL Oisterwijk, The Netherlands; alveusab@outlook.com (J.S.)
* Correspondence: ichinou@pharm.uoa.gr

SUPPLEMENTARY MATERIALS

Table S1. Chemical composition (% Relative abundance) by GC-MS analysis of propolis samples from different geographic regions of Latvia

		Relative abundance %									
**RT(min)**	**Compound**	**LV01***	**LV02***	**LV03***	**LV04***	**LV05***	**LV06***	**LV07***	**LV08***	**LV09***	**LV10***
5.33	benzoic acid	23.61	23.53	22.24	29.63	31.41	27.80	33.04	24.98	33.01	30.88
6.10	phosphoric acid	1.15	1.14						1.49		2.08
7.70	*p*-hydroxybenzaldehyde		0.59					0.81			
8.42	hydroquinone	0.51	0.73	0.56			0.74	0.72			0.62
10.56	malic acid	0.36									
11.06	vanillin	3.49	8.02	4.74	5.65	9.24	10.44	9.42	5.59	2.94	6.60
11.26	cinnamic acid	2.52	0.57	8.19	0.47	0.51	0.47	1.07		0.51	0.72
12.04	caryophyllene oxide				0.48					0.55	
12.37	2,3,4-trihydroxybutyric acid								0.44		
13.07	δ-selinene	0.53		0.76							
13.18	4-hydroxybenzoic acid	1.01	1.36	0.65	1.48	0.73	1.14	0.84	1.09	1.45	1.17
13.44	β-eudesmol	0.77		0.94							
13.56	α-eudesmol	1.74	1.52	1.24		2.53					
13.70	dodecanoic acid	0.43	0.32	0.41			0.30				0.53
15.09	pentanedioic acid		0.23		0.39		0.37	0.41		0.55	0.34
15.57	dehydroaromadendrene								0.31	0.60	
15.78	benzyl benzoate	<0.1	0.59	0.58	1.45	<0.1	2.71	1.37	0.87	1.81	0.56
16.08	4-hydroxy-3-methoxy benzoic acid	0.22	0.36	0.46	0.31	0.38	0.47	0.40			0.29
17.00-18.71	sugars	19.57	26.70	17.59	26.98	19.43	15.80	17.83	32.63	17.83	22.87
16.53	cis-*p*-coumaric acid	1.42	0.74	0.95	0.53	0.81	0.81	0.70	0.80	1.05	0.89
16.70	*p*-coumaryl alcohol	0.98	0.38	0.89	0.27		0.72	0.65			0.39
17.45	coniferyl aldehyde				0.62	0.77	1.06	1.22			
17.70	myristic acid			0.35	<0.1		0.62				0.51

19.16	*Z*-ferulic acid	0.59	0.55	0.48	0.43	0.65	0.51	0.43	0.34	0.63	0.62
19.22-19.81	sugars	1.86	4.62		3.25	0.63	0.36	2.18	2.93	1.95	2.58
19.48	trans-*p*-coumaric acid	13.82	11.88	14.00	8.07	11.91	11.46	10.17	10.99	11.55	11.73
20.88-21.14	sugars	1.04	2.91	2.26	4.80	4.28	3.88	3.47	4.79	3.19	2.03
21.34	3-hydroxymyristic acid	0.36									
21.43	palmitic acid	1.13	1.49	1.30	0.92	1.41	1.76	1.05	1.41	0.63	1.52
21.98	sugar		0.27								
22.04	isoferulic acid	0.36									
22.17	benzyl cinnamate	0.33	<0.1	0.37	<0.1	<0.1	1.20	1.48	<0.1	<0.1	<0.1
22.31	*E*-ferulic acid	5.50	5.47	5.30	3.73	5.98	6.36	5.86	3.01	4.57	4.93
23.00	sugar	0.26		0.60							
23.24	caffeic acid	3.06	0.93	2.74	0.50	0.93	1.30	1.00	0.50	0.84	0.90
24.02	pentenyl-*p*-coumarate	0.36		0.65							
24.28	linoleic acid	0.32									
24.39	oleic acid	0.60									
24.68	3-hydroxypalmitic acid	0.25									
26.79	3-methyl-3-butenyl caffeate	1.31									
27.48	2′,6′-dihydroxy-4′ methoxy dihydrochalcone	0.80		1.12							
27.64	3-methyl-2-butenyl caffeate	0.82		0.29							
28.15	2′,4′,6′-trihydroxy-dihydrochalcone	0.30		0.80							
28.42	benzyl ester derivative						0.42	0.39			
28.84	pinostrobin chalcone	0.36		1.33							
28.99	benzyl-*p*-coumarate	2.14	0.91	1.57	0.51	1.33	2.42	1.48	0.54	1.83	1.16
29.20	pinostrobin			<0.1							
29.45	pinocembrin	1.02	0.17	1.11							
30.43	pinobanksin	1.00		0.54							
30.43	sugar		0.43		0.37	0.72					0.38
30.81	coniferyl benzoate	0.89	1.41	1.01	1.07	2.24	3.30	3.23	0.64	0.40	1.19
30.99	cinnamyl cinnamate			<0.1							
31.09	2′,6′-dihydroxy-4,4′ dimethoxy dihydrochalcone	<0.1		<0.1							
31.41	benzyl ferulate						0.55				
31.57	pinobanksin 3-*O*-acetate	0.50		0.53							
31.60	trihydroxy-methoxy dihydrochalcone			<0.1							
32.12	sugar		0.25								
32.25	trihydroxy-methoxy dihydrochalcone	<0.1		<0.1							
32.70	galangin	0.83		0.44							

*Propolis samples from different geographic regions of Latvia: LV01: Jelgava, LV02: Riga, LV03: Daugavpils, LV04: Rujiena, LV05: Balvi, LV06: Aizpute, LV07: Svete, LV08: Ragana, LV09: Talsi, LV10: Cesis.
